# Bird flu outbreak amidst COVID‐19 pandemic in South Africa: Efforts and challenges at hand

**DOI:** 10.1002/jmv.27124

**Published:** 2021-06-14

**Authors:** Olivier Uwishema, Lubanga F. Adriano, Elie Chalhoub, Helen Onyeaka, Melissa Mhanna, Success C. David, Yves Nasrallah, Lucas L. P. A. Ribeiro, Christin Berjaoui

**Affiliations:** ^1^ Oli Health Magazine Organization Research and Education Kigali Rwanda; ^2^ Clinton Global Initiative University New York New York USA; ^3^ Faculty of Medicine Karadeniz Technical University Trabzon Turkey; ^4^ University of Malawi College of Medicine Blantyre Malawi; ^5^ Faculty of Medicine University of Saint Joseph of Beirut Beirut Lebanon; ^6^ School of Chemical Engineering University of Birmingham Edgbaston Birmingham B152TT UK; ^7^ Faculty of pharmaceutical Sciences University of Nigeria Nsukka Enugu State Nigeria; ^8^ School of Medicine and Medical Sciences Holy Spirit University of Kaslik Beirut Lebanon; ^9^ School of Medicine University of Fortaleza Fortaleza Ceará Brazil; ^10^ Faculty of Medicine Beirut Arab University Beirut Lebanon

**Keywords:** Africa, avian influenza, bird flu, COVID‐19, influenza pandemic, outbreak, South Africa

## Abstract

Over the months of April and May 2021, South Africa has witnessed several outbreaks of highly infective avian influenza (H5N1) in different poultry farms. This came as a shock to a country that was already battling with the deadly COVID‐19 pandemic. The emergence of the virus has spurred import bans and massive culls in the poultry business. Local experts have also called for a restriction on the movement of people and cars in and out of their chicken farms. Employees have also been encouraged to shower in the mornings when they arrive at the farms and wear fresh clothes, as the flu spreads very quickly. In a country that is already facing the economic implications of the COVID‐19, this has the potential to cause a significant dent in the economy, as well as severely impact people's day‐to‐day life. Bird flu—also called avian influenza—is a viral infection that can infect not only birds but also humans and other animals. The threat of a new influenza pandemic has prompted countries to draft national strategic preparedness plans to prevent, contain and mitigate the next human influenza pandemic. This paper describes the South African burden, current efforts, and preparedness against the avian influenza virus.

## 1 INTRODUCTION

In 2005, the World Health Organization (WHO) labeled the avian influenza viruses (AIVs)—which attack mainly bird species—as one among the nonprioritized zoonosis.^1^ Even though high‐ranked international organizations recommended that more efforts be made in fighting these diseases,^2^, ^3^, ^4^, ^5^ little has been done as they remain neglected.^1^ However, AIVs impose high morbidity and mortality risks,^6^, ^7^ and cause important economic losses.^8^ For these reasons, more focus should be diverted to Africa as it already suffers from the burden of different infectious diseases, a weak healthcare system, and more reliance on poultry as a food source and a business industry.^9^, ^10^

On the other hand, as AIVs are impossible to eliminate, rigorous surveillance should be implemented to investigate suspicious outbreaks so that the risk could be minimized.^11^ Nevertheless, sub‐Saharan Africa (SSA) has difficulties managing zoonosis due to multiple factors of which we cite finance insufficiency, inappropriate facilities, and lack of biosecurity measures.^2^, ^12^ Consequently, despite all the conducted efforts, the highly pathogenic avian influenza (HPAI) H5N1 remains in different parts of the African ecosystems.^13^ Even worse, the coronavirus disease 2019 (COVID‐19) pandemic added a huge burden on the emerging African nations, leaving them in a more disadvantageous situation.^14^

By May 12, South African regulators reported nine HPAI H5 outbreaks in a monthly period, occurring in different parts of the country.^15^ Some had different strains,^15^ which means that they have separate causes. One of these outbreaks was the H5N8 type, while all the remaining others the H5N1 type. Moreover, a similar H5N8 outbreak was detected in 2017^14^; and even though it was believed that H5N8 does not infect humans, the Russian health department proved the opposite in February 2021.^16^ Furthermore, following the latest update of the H5N1 status in Nigeria, the country's Center for Disease Control declared that they had detected seven patients infected by influenza A (H5) by March 2021,^17^ and the latest reported case in all Africa was in 2007.^17^ Although H5N1's human‐to‐human transmission is infrequent, the WHO fears a genetic mutation that could make this phenomenon possible; hence, inflicting new global health and economic crises^18^ equivalent to the new COVID‐19 pandemic.

Considering the lack of HPAI management policies in the SSA in general,^2^, ^12^ and South Africa in particular,^14^ the One Health program led by the WHO to fight zoonosis is harder to be achieved.^2^ Therefore, it is essential to review the status of the AIVs before and during COVID‐19 in South Africa and assess the challenges and efforts essential to mitigate the additional burden of bird flu in this nation.

## 2 BURDEN AND CURRENT STATUS OF BIRD FLU IN SOUTH AFRICA DURING COVID‐19 PANDEMIC

The SARS‐CoV‐2 virus, which emerged in late 2019 based on the COVID‐19 pandemic, has caused a global health emergency leading to a state of alertness from all the nations against this single virus. Meanwhile, during April and May 2021, South Africa has witnessed several outbreaks of avian influenza (H5) in different poultry farms.^19^ The H5 strain is responsible for the HPAI that caused several epidemics throughout the years, the last one in 2017.^20^ However, according to the Food and Agriculture Organization, there is no report of low pathogenic avian influenza since April 28, 2021.^21^

According to the WHO, there has been no contamination to human yet^22^; however, the South Africa Poultry Association confirms that all measures of precautions are being taken in the concerned farms: farms' isolation, culling of the exposed birds… to prevent a potential outbreak amid the COVID‐19 pandemic which can make the situation hard to control.^23^ Due to the numerous similarities between Influenza and COVID‐19 in terms of the symptoms—fever, cough, shortness of breath; the vulnerable population—elderly population (>65 years old); the incubation period—2–14 days for COVID‐19 and 2–5 days for influenza; the way of transmission—respiratory droplets,^24^, ^25^ the differentiation between the two viruses would be difficult accounting for a possible delay in diagnosis, therefore increase in hospitalization, ICU admission, and risk of an increased mortality rate (Table 1). It is worth noting that the five regions hit by the HPAI outbreak constitute around 60% of the total COVID‐19 cases in South Africa.^26^ Two of them, Gauteng and Western Cape, are facing the biggest burden as the numbers of infections makeup, respectively, 26.8% and 18.1% of the total incidents.^26^

**Table 1:** Comparison between COVID‐19 and avian influenza/bird flu

	COVID‐19	Avian influenza
Common symptoms	Fever, cough, shortness of breath	Fever, cough, shortness of breath
Most affected age group	>65 years old	>65 years old
Incubation period	2–14 days	2–5 days
Way of transmission	Respiratory droplets	Respiratory droplets

*Note*: Those similarities make the differentiation between the two viruses hard.

Some African countries have already stopped importing poultry products from South Africa as a preventive measure^27^; therefore, more than 1300 people have lost their jobs, and South Africa is witnessing an increase in prices of poultry products because of the preventive measures farms are taking which are making those products less affordable for lower‐income groups. All countries, including South Africa, are already struggling economically during the COVID‐19 pandemic; thus, the country cannot tolerate an added crisis due to the avian influenza.^28^

## 3 CURRENT EFFORT IN RESPONSE TO BIRD FLU IN SOUTH AFRICA AND CHALLENGES FACED DURING COVID‐19 PANDEMIC

Bird flu—also referred to as avian influenza—is an influenza A virus. It is fatal to poultry and is potentially destructive in humans. Bird flu spreads between both wild and domesticated birds. It has also been passed from birds to humans in close contact with poultry or other birds.^29^

The impact of COVID‐19 on the economy and then the concomitant disruptive effect of the bird flu outbreak has a rough impact on the employment and welfare of the country.^30^ Humans in contact with the contaminated bird are at risk of developing health problems that can be detrimental amid the COVID‐19 pandemic that has overwhelmed the healthcare system. Furthermore, the outbreak of bird flu amid the present ongoing COVID‐19 pandemic has affected people psychologically due to fear and anxiety. Again, it may result in another lockdown of the nation and thereby affect vaccination of the country for COVID‐19, which could become another hazard.

It was reported by Meyer on April 20, 2021 that the whole poultry sector is now on alert and biosecurity contingency plans have been set in place. Also, he underlined the necessity to report any sick or dead birds to the veterinary services. Poultry producers are advised to step up their biosecurity to avoid virus contamination from the wild birds or their stools.^30^

Therefore, WHO has pointed out the need for collaboration between the animal and public health sectors to identify disease activity in animals to control the diseases in animals regarding reduced human exposure.^31^ There should be an effective means of communication to the rural resident to improve their understanding of animal diseases and human behavior, and the possible risk of acquiring avian influenza.^31^ Also, methods to early detection of viruses should be better understood for conditions that predispose humans to infection and emergence of a pandemic.^31^

## 4 RECOMMENDATIONS

The COVID‐19 pandemic has highlighted different challenges related to healthcare across the world, such as accessibility, awareness, the importance of evidence‐based practices, and trustworthy information for the public, and also the risk of rising other highly infectious diseases such as HPAI. The HPAI is a worrying disease in public and global health, but until this moment, there have not been cases of the H5 strain of HPAI causing an infection in humans in the South African outbreaks in April and May of 2021.^15^

As those outbreaks were recent, very few studies have been conducted regarding its manifestations, specificities, infection ratio, and possible infection in humans. In this context, there is a demand for evidence‐based data, with a highlight on epidemiological studies, it is necessary to conduct further studies on HPAI in the scenario of the global pandemic of SARS‐CoV‐2.^15^, ^22^

One health concept is important in HPAI disease, demanding awareness from governmental powers, interdisciplinary work to prevent food and animal infections, and a structured action plan to prevent mass casualties.^32^

In this context, the authors recommend adopting interdisciplinary initiatives, highlighting the need for collaboration with experts on animal and environmental health between the local and national health workforce of the region.^32^, ^33^ Collaboration is imperative to minimize damages, prevent infections, provide quality healthcare for the population, and develop a safe environment for all people.

## 5 CONCLUSION

The emergence of bird flu, amidst the COVID‐19 pandemic in South Africa, is another threat to the economy and public health of the people in the country. In a country that has already suffered huge losses due to the COVID‐19 pandemic, the fight against the new zoonoses may not be as effective as necessary. The country's efforts are channeled towards the earlier pandemic, and this may lead to neglect of the flu, which could also have another disastrous effect on the economy. Though there is no reported case of human infection, the country's health system needs to be vigilant, and establish a good preparedness system that would be able to deal with such outbreaks. The country also needs to institute a strong surveillance system for the mitigation of the flu. Farmers that have suffered huge losses because of the flu need to be supported. This calls for strong collaboration between the government and other stakeholders.

## CONFLICT OF INTERESTS

The authors declare that there are no conflict of interests.

## AUTHOR CONTRIBUTIONS

*Conceptualization, project administration, writing—review and designing*: Olivier Uwishema. *Supervised, reviewed, and edited the first and second draft*: Helen Onyeaka. *Collection and assembly of data*: Olivier Uwishema, Lubanga F. Adriano, Elie Chalhoub, Helen Onyeaka, Melissa Mhanna, Success C. David, Yves Nasrallah, Lucas L. P. A. Ribeiro, and Christin Berjaoui. *Data analysis and interpretation*: Olivier Uwishema, Lubanga F. Adriano, Elie Chalhoub, Helen Onyeaka, Melissa Mhanna, Success C. David, Yves Nasrallah, Lucas L. P. A. Ribeiro, and Christin Berjaoui. *Manuscript writing*: Olivier Uwishema, Lubanga F. Adriano, Elie Chalhoub, Helen Onyeaka, Melissa Mhanna, Success C. David, Yves Nasrallah, Lucas L. P. A. Ribeiro, and Christin Berjaoui. *Final approval of manuscript*: Olivier Uwishema, Lubanga F. Adriano, Elie Chalhoub, Helen Onyeaka, Melissa Mhanna, Success C. David, Yves Nasrallah, Lucas L. P. A. Ribeiro, and Christin Berjaoui.
